# Starvation causes changes in the intestinal transcriptome and microbiome that are reversed upon refeeding

**DOI:** 10.1186/s12864-022-08447-2

**Published:** 2022-03-22

**Authors:** Jayanth Jawahar, Alexander W. McCumber, Colin R. Lickwar, Caroline R. Amoroso, Sol Gomez de la Torre Canny, Sandi Wong, Margaret Morash, James H. Thierer, Steven A. Farber, Brendan J. M. Bohannan, Karen Guillemin, John F. Rawls

**Affiliations:** 1 Department of Molecular Genetics and Microbiology, Duke Microbiome Center, Duke University School of Medicine, Durham, NC 27710 USA; 2 Department of Civil and Environmental Engineering, Duke University, Durham, NC 27708 USA; 3 Department of Evolutionary Anthropology, Duke University, Durham, NC 27708 USA; 4 Department of Embryology, Carnegie Institution for Science, Baltimore, MD 21218 USA; 5 Department of Biology, Johns Hopkins University, Baltimore, MD 21218 USA; 6 Institute of Ecology and Evolution, University of Oregon, Eugene, OR 97403 USA; 7 Institute of Molecular Biology, University of Oregon, Eugene, OR 97403 USA

## Abstract

**Background:**

The ability of animals and their microbiomes to adapt to starvation and then restore homeostasis after refeeding is fundamental to their continued survival and symbiosis. The intestine is the primary site of nutrient absorption and microbiome interaction, however our understanding of intestinal adaptations to starvation and refeeding remains limited. Here we used RNA sequencing and 16S rRNA gene sequencing to uncover changes in the intestinal transcriptome and microbiome of zebrafish subjected to long-term starvation and refeeding compared to continuously fed controls.

**Results:**

Starvation over 21 days led to increased diversity and altered composition in the intestinal microbiome compared to fed controls, including relative increases in *Vibrio* and reductions in *Plesiomonas* bacteria. Starvation also led to significant alterations in host gene expression in the intestine, with distinct pathways affected at early and late stages of starvation. This included increases in the expression of ribosome biogenesis genes early in starvation, followed by decreased expression of genes involved in antiviral immunity and lipid transport at later stages. These effects of starvation on the host transcriptome and microbiome were almost completely restored within 3 days after refeeding. Comparison with published datasets identified host genes responsive to starvation as well as high-fat feeding or microbiome colonization, and predicted host transcription factors that may be involved in starvation response.

**Conclusions:**

Long-term starvation induces progressive changes in microbiome composition and host gene expression in the zebrafish intestine, and these changes are rapidly reversed after refeeding. Our identification of bacterial taxa, host genes and host pathways involved in this response provides a framework for future investigation of the physiological and ecological mechanisms underlying intestinal adaptations to food restriction.

**Supplementary Information:**

The online version contains supplementary material available at 10.1186/s12864-022-08447-2.

## Introduction

Starvation is a state of severe caloric restriction regularly experienced by many animal species and a significant portion of the human population. In humans, starvation can be the result of environmental or socioeconomic conditions including war, famine, and poverty [1]. It can also occur alongside pathologies such as anorexia nervosa and cancer [2]. In animals, periods of absolute or relative starvation can result from seasonal changes such as drought and severe cold, or from behaviors such as nesting, lactation, migration, and hibernation [3]. This wide range of circumstances leading to starvation across the animal kingdom evokes a range of progressive physiologic adaptations to starvation across different species. Indeed, previous studies have reported similarity and divergence in starvation physiology across animal taxa such as humans, rodents, polar bears, penguins, reptiles, amphibians, fish, and insects [4]. However, previous studies have largely focused on tissue histopathologies associated with starvation, whereas effects on the underlying physiological processes remain incompletely understood.

Across many animal species, starvation leads to a progressive decrease in metabolic rate [5]. Increased blood glycerol, which serves as a gluconeogenic precursor, is also common in starved animals, as are fluctuations in free fatty acids [3, 4]. The overall depletion in energy stores leads to weight loss, which is generally greater in endotherms when compared to ectotherms [6]. Starvation is also associated with a gradual reduction in mass in important organs such as the liver, skeletal muscle, and intestine [4, 7]. These effects necessitate a recovery from starvation to restore optimal function to these organs. Inherently linked to starvation, the return to homeostasis following starvation is facilitated by a refeeding response that gradually reverses starvation-induced adaptations and restores energy balance. Physiological responses to starvation and subsequent refeeding are dynamic and complex, involving coordination between major organ systems via nutritional and hormonal signals. The ultimate outcome of these starvation responses is often the preservation of lean body mass while favoring the depletion of energy stores such as glycogen and fat [8, 9]. However, despite these effects, starvation often results in lasting defects on bone density, pancreatic function, and mental development long after refeeding [10–12]. Thus, improved understanding of these dynamic physiological processes could lead to new approaches to reduce morbidities and mortalities associated with starvation in humans and other animals [13].

Previous studies on the effects of refeeding after starvation have largely focused on tissues such as liver, skeletal muscle, brain, and pancreatic islets [14–18]. We have a relatively poor understanding of the transcriptional starvation and refeeding responses in the intestine. The intestine is the major site of dietary nutrient sensing and absorption, and harbors complex communities of microorganisms (microbiome). Previous studies in humans and rodent models have shown that intestinal microbiome composition changes in response to starvation and diet composition with distinct contributions to the nutritional physiology of their hosts [19–26]. These findings informed more recent studies that have investigated microbiome-targeted therapeutics for alleviating starvation and its associated developmental defects [27–29]. However, gut microbial responses to starvation have been largely limited to mammals, and our understanding of intestinal physiological responses to starvation and feeding in any animal remains quite limited.

Animal models provide opportunities to study the processes that underlie starvation and refeeding responses in vertebrates, resulting in a general understanding that may be translated to humans [30]. Poikilothermic vertebrates such as cyprinid fishes are particularly interesting due to their capacity to endure prolonged starvation periods. In response to prolonged starvation, cyprinids such as carp exhibit a reduction in intestinal thickness and weight, altered enterocyte morphology, and a decrease in body weight and liver size, similar to the starvation response in mice [17, 31–35]. Zebrafish (*Danio rerio*) survive up to 4 weeks of starvation as adults, and a suite of genomic and genetic resources facilitate the investigation of their physiology [36]. Using in vivo imaging to monitor white adipose tissues as a measure of energy storage, we previously showed that prolonged starvation in adult zebrafish leads to progressive mobilization of fat stored in white adipose tissues, which is replenished in response to refeeding [37–39]. Because adipose tissues develop progressively during juvenile and adult stages, the duration of starvation required to completely mobilize adipose lipid increases with animal age (e.g., from 1 week in juveniles up to 3 weeks in adults) [37–39]. However, the impact of prolonged starvation and refeeding on the zebrafish intestine has not been explored.

The zebrafish intestine displays extensive cellular and physiological homology to that of mammals, and harbors a microbiome that varies in composition as a function of age and diet composition [40–45]. The presence and composition of the intestinal microbiome in zebrafish impacts the host by regulating dietary nutrient absorption, epithelial renewal, and inflammation [41, 43, 46–50]. By comparing patterns of gene expression and accessible chromatin in intestinal epithelial cells from zebrafish, mouse, and human, we recently discovered a conserved transcriptional regulatory network conserved across 420 million years of vertebrate evolution [51]. Building upon this recent work, here we define the impact of prolonged starvation and refeeding on gene expression in the adult zebrafish intestine, and use these results to predict the physiological processes and transcription regulatory pathways that underlie the response to starvation and refeeding. We also show how the taxonomic composition of the adult zebrafish intestinal microbiome is altered during the same prolonged starvation and refeeding regimen.

## Results

### Starvation is accompanied by significant changes in gut microbiome composition that are reversed during refeeding

**Fig. 1:**
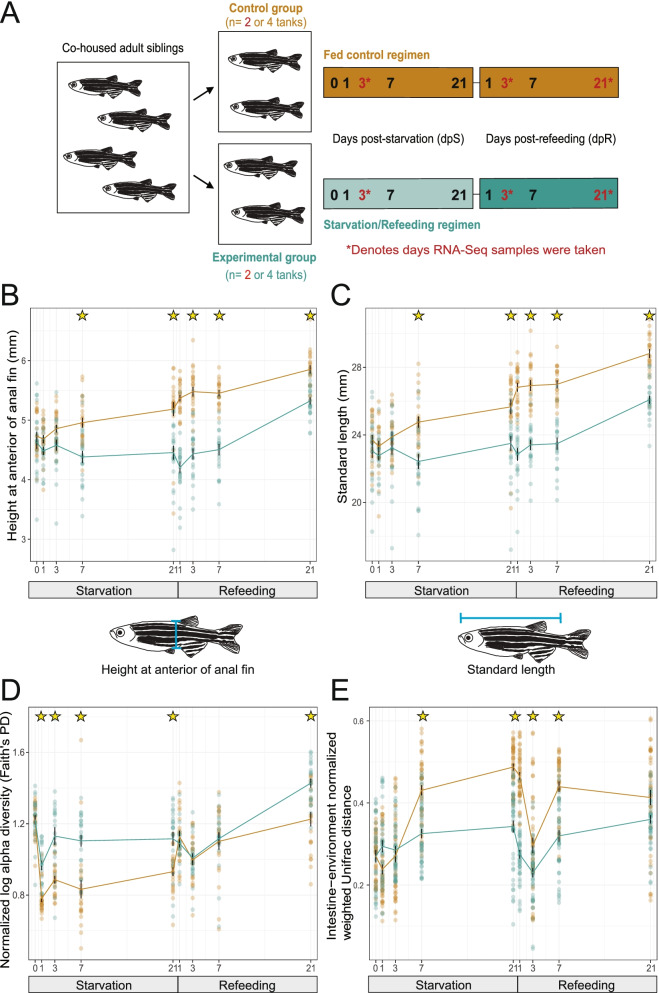
Starvation and refeeding affect zebrafish somatic growth as well as intestinal and environmental microbiome diversity. **A** Study design schematic. Cohoused adult siblings were divided into either control (fed) or experimental (starved) tanks. Samples were then taken from each tank on days 0, 1, 3, 7 and 21 post-starvation (dpS) as well as 1, 3, 7, and 21 days post-refeeding (dpR) for 16S rRNA gene sequencing. RNA-seq samples were taken at 3 dpS, 21 dpS, and 3 dpR. **B** Fed and starved zebrafish height at anterior of anal fin (HAA) in mm at corresponding timepoints. **C** Standard length in mm of starved and fed zebrafish. **D** Faith’s PD alpha diversity for fed and control zebrafish. Values are log transformed and normalized by the scores at day 0. **E** Weighted UniFrac distance between the gut and associated environment sample. Stars in panels B-E denote significant difference (*p* < 0.05 by Tukey’s HSD test)

To determine the influence of starvation on zebrafish microbiome composition and intestinal gene expression, zebrafish were reared under conventional conditions using a standard diet to early adulthood (60 days post fertilization or dpf). Animals were then moved into clean tanks and randomly assigned into one of two treatment groups: one group was starved for 21 days followed by 21 days refeeding, and a reference control group was consistently fed across the same 42-day time course (Fig. 1A). This 21 day starvation regimen was selected because it is sufficient in adult zebrafish to completely deplete stored lipid from adipose tissues and reduce body weight and liver size, whereas subsequent refeeding largely restores total adipose tissue lipid, body weight, and liver size within 14 days [37, 38, 52, 53]. We performed 16S rRNA gene sequencing on whole intestinal samples from zebrafish at 0, 1, 3, 7, and 21 days post-starvation (dpS), then at 1, 3, 7, and 21 days post-refeeding (dpR) with a standard diet (Fig. 1A). Age matched siblings fed the same standard diet on a daily basis served as reference controls and were sampled at the same time points.

During these early adult stages, zebrafish fed normally continued to display somatic growth as expected. Measurements of animal size as standard length (SL) and height at anterior anal fin (HAA) revealed that somatic growth in starved fish was largely arrested compared to control fish (Fig. 1B-C). Starved fish were significantly smaller than fed fish by 7 days post-starvation and this trend continued beyond the end of the starvation period. Starved animals resumed growth after refeeding, though they remained significantly smaller than fed fish throughout the duration of the experiment (*p* < 0.05, two-way ANOVA with Bonferroni correction) (Fig. 1B-C). We observed no mortality in any of these conditions consistent with previous studies [38, 53]. Starvation therefore caused a general arrest in somatic growth which was restored upon refeeding.

**Fig. 2:**
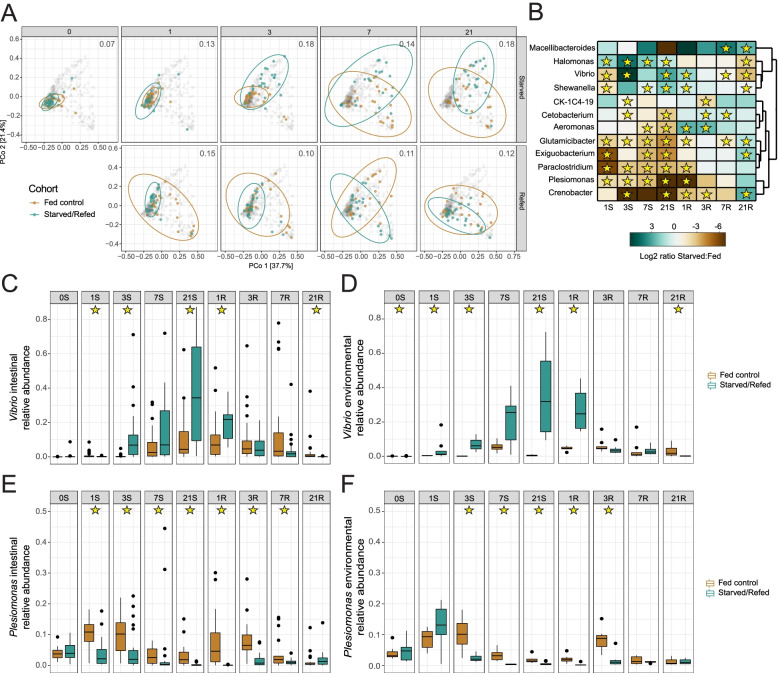
Starvation and refeeding dynamically alters composition of the adult zebrafish intestinal microbiome. **A** Principal coordinates analysis of weighted UniFrac diversity for fed and starved zebrafish. The distance between centroids of the two cohorts at the corresponding timepoint is shown in the top right of each plot. The gray dots represent every sample in the study, while the samples from a given timepoint are labeled in their respective panel according to their cohort (green: starved/refed, gold: fed controls). **B** Heatmap of log2 ratio of the relative abundance of bacterial genera between starved and fed controls. Stars denote day identified as significant by LEfSe. **C** Relative abundance of *Vibrio* in starved and control zebrafish intestines by day. **D** Relative abundance of *Vibrio* in starved and control environmental tank water samples by day. **E** Relative abundance of *Plesiomonas* in starved and control zebrafish intestines by day. **F** Relative abundance of *Plesiomonas* in starved and control environmental tank water samples by day. Stars in panels C-F denote significance (*p* < 0.05) by pairwise Wilcoxon test with BH correction

Analysis of 16S rRNA gene sequencing data from intestinal samples revealed the impacts of prolonged starvation and refeeding on intestinal microbiome composition. Overall the intestinal microbiomes of starved zebrafish maintained a higher Faith’s PD diversity compared to fed controls. Both conditions displayed an initial loss of diversity by 1dpS, perhaps due to stress caused by tank transfer at 0dpS when the experiment began. However starved communities maintained significant higher diversity from 1dpS through the end of starvation at 21dpS and again at 21dpR (*p* < 0.05, ANOVA and Tukey HSD) (Fig. 1D). Beta diversity analysis of community composition using weighted UniFrac distances showed that starved and fed communities began to differ by 1 dpS, with the centroid distances being greatest at 3dpS and 21 dpS (Fig. 2A). During refeeding, the starved fish samples quickly returned to a composition more similar to fed controls (Fig. 2A). PERMANOVA further confirmed that starvation, experimental time point, and the interaction are significant factors (*p* < 0.05, *R*^2^ = 0.017, 0.17 and 0.07, respectively) affecting gut microbiome composition. Thus, prolonged starvation induced detectable shifts in overall composition of gut bacterial communities that were reversed quickly after refeeding.

### Starvation increases similarity between zebrafish gut microbiomes and their surrounding water environment

The microbiome residing in the zebrafish intestine exists in continuity with that of the surrounding water environment, however these communities typically display distinct compositions [42–44]. The ecological processes contributing to these differences remain unclear, but could include non-neutral processes such as host selection [54, 55] or the magnitude of dispersal between the intestine and the surrounding environment [56]. To test if starvation and refeeding alter the relationship between the gut and environmental microbiomes, we compared weighted UniFrac distance between matched gut and environmental samples in starved/refed and control fish normalized by the day 0 values. The distance between gut and environmental samples increased between 0 dpS and 21 dpS (Fig. 1E), perhaps reflecting restoration of homeostasis after the stress of transfer into new tanks that occurred at 0 dpS. However, from 7 dpS to 7 dpR the distance between gut and environmental samples was greater for starved than fed controls. This suggests that the fed controls had an intestinal microbiome composition more similar to the environment compared to starved fish during those stages. At 21 dpR these distances were not statistically different between treatment groups. These results indicate that starvation increases similarity between gut and environmental communities but refeeding restores differences between these communities to levels achieved under constant feeding conditions.

### *Vibrio* bacteria are significantly enriched in the intestine during starvation

We next sought to identify the specific bacterial taxa that were significantly affected by starvation and refeeding using LEfSe [57]. LEfSe identified 120 genus-level taxa that reached a logarithmic LDA score of 2.0 (Table S1). This set of affected taxa included 12 abundant genera (median relative abundance > 0.1% across all samples; shown in Fig. 2B) including starvation-induced depletion of *Plesiomonas* and enrichment of *Vibrio*. Strikingly, *Vibrio* reached a maximum relative abundance of 87% (median 34%) in the intestines of starved zebrafish at 21dpS, which was markedly higher than that of fed controls (maximum 62%, median 4%) at the same time point (Fig. 2C). In contrast, starvation led to reduced relative abundance of *Plesiomonas* in the intestine by 1dpS continuing through 7dpR (Fig. 2E). These effects of starvation on *Vibrio* and *Plesiomonas* sp. in the starved guts were reflected in the environmental samples of the starved fish (Fig. 2D-F). Importantly, none of the phyla or orders that were significantly depleted or enriched were significantly correlated with SL after Bonferroni correction (see Table S2). This suggests that their depletion and enrichment are due to the dietary treatment, and not simply the growth arrest observed in starved animals (Fig. 1C-D). These results establish that prolonged starvation leads to significant alterations in intestinal microbiome composition including marked enrichment of *Vibrio* genus members, and that these alterations in microbiome composition are largely normalized within 1–3 days of refeeding [41, 42, 48].

### Starvation and refeeding leads to distinct changes in intestinal gene expression that vary with the duration of starvation

**Fig. 3:**
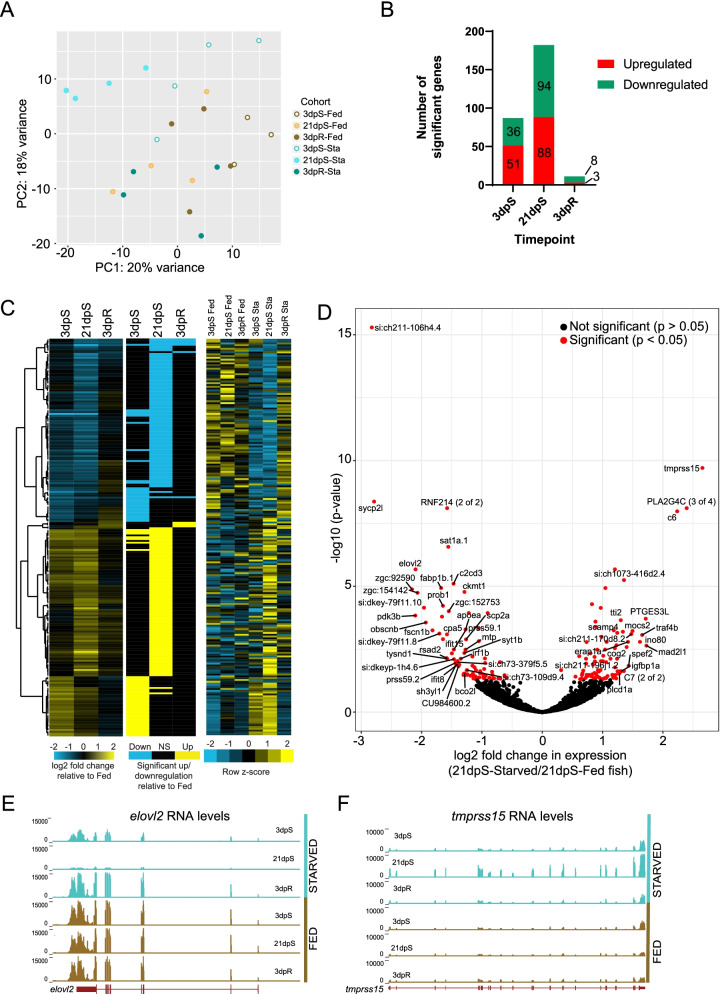
Starved zebrafish differentially regulate intestinal gene expression when compared to fed zebrafish. **A** Principal Components Analysis (PCA) of RNA-Seq libraries in starved/refed and fed control zebrafish intestines at 3dpS, 21dpS, and 3dpR. **B** Quantification of the number of significantly upregulated and downregulated genes in starved/refed zebrafish intestines at each timepoint. Note that these numbers in panel B include genes that were also significantly differential in our fed control comparisons. **C** Hierarchical clustering of log2 fold changes in gene expression in starved zebrafish intestines, along with flattened values that show significant changes in gene expression, and z-scores based on normalized counts of each gene. **D** Log2 fold changes in gene expression in starved zebrafish intestines at 21dpS when compared to 21dpS fed fish plotted according to their -log_10_ adjusted *p*-values. Note that data plotted in panels C and D do not include genes that were significantly differential within fed fish (see Table S3B and Fig. S2). **E** UCSC tracks of representative replicates show that *elovl2* mRNA, encoding a fatty acid elongase, is downregulated in starved zebrafish intestines and returns to levels comparable to the fed group upon re-feeding. **F** UCSC tracks of representative replicates show that *tmprss15* mRNA, encoding an enteropeptidase, is upregulated in starved zebrafish intestines and returns to levels comparable to the fed group upon re-feeding

Previous work in vertebrates has shown that starvation can significantly affect host gene expression in multiple organs [4, 7, 17, 33, 52]. Our analysis of intestinal microbiomes during starvation and refeeding suggested distinct stages - early starvation when microbiome effects are initially observed (i.e., 3 dpS), late starvation when microbiome alterations are greatest (i.e., 21 dpS), and early refeeding when microbiome composition is largely normalized (i.e., 3 dpR). We therefore dissected whole intestinal tracts from 3dpS, 21dpS, and 3dpR adult zebrafish and their fed age-matched controls for RNA-seq analysis (3–4 biological replicate samples/condition; Fig. 1A). Principal components analysis (PCA) of these data revealed similarities between biological replicates (Fig. 3A). Similar to the observed effects on the intestinal microbiome, PCA indicated the impact of starvation on intestinal gene expression was greater at 21dpS than 3dpS or 3dpR. We then used DEseq2 analysis to identify genes differentially expressed in starved/refed fish compared to their fed controls at each timepoint (Table S3A). In accord with our PCA analysis, the number of significant differentially expressed genes increased from 87 genes at 3dpS to 182 genes at 21dpS, and was reduced to 11 genes by 3dpR (Fig. 3B). This further supports that starvation has a progressive impact on the intestinal transcriptome through 3dpS and 21dpS which is largely normalized by 3dpR.

As a control and to estimate the influence of the developmental time covered during the experiment, we compared differential gene expression between the fed timepoints. There were 135 genes found to be significant in these comparisons, but only 30 of these were represented among significant genes in our Starved vs. Fed comparisons (18 at 3dpS, 15 at 21dpS, 2 at 3dpR; listed in Table S3B and shown in Fig. S1B) and they were removed from our subsequent analyses of starvation effects (see Fig. S2 for results with those genes retained in the analysis). Hierarchical clustering of the log_2_ fold changes in transcript abundance revealed distinct groups of genes that were upregulated or downregulated in response to starvation, including striking differences between the response to starvation at 3dpS and 21dpS (Fig. 3C). Though gene expression differences between starved/refed fish and fed controls was largely restored by 3dpR, there was a small set of 9 genes that continued to be differentially expressed even at 3dpR (Fig. 3B; Table S3A). Although this list of persistent genes was too small to permit functional enrichment analysis, it does suggest a potentially small group of intestinal functions that remain altered after restoration of feeding or that respond to both starvation as well as to refeeding. These persistently different genes are discussed below in context.

Among the genes known to be starvation responsive, we first examined transcript levels of *elovl2*, a fatty acid elongase previously shown to be downregulated in zebrafish during starvation [58–60]. Elovl2 has also been implicated in inducing insulin secretion in response to glucose in mice, and fatty acid elongases have been extensively studied in fish as they function in biosynthesis of long-chain polyunsaturated fatty acids, which are commercially important in fish aquaculture [58, 61, 62]. In accord, *elovl2* was significantly downregulated by 3dpS, was one of the most significantly downregulated genes in starved fish at 21dpS, and was also consistently expressed across the control fed fish group (Fig. 3D-E, Fig. S1A). This downregulation suggested a reduction in intestinal fatty acid synthesis during starvation.

To understand which biological processes are impacted by starvation, we performed Gene Ontology (GO) term searches of four groups of genes from our dataset; genes significantly upregulated at 3dpS or 21dpS, and genes significantly downregulated at 3dpS or 21dpS (Figs. 3C, S1C-D, and S3). We first identified distinct, non-overlapping functions that were enriched early in starvation (i.e., at 3dpS) and late in starvation (i.e., at 21dpS). For example, functions enriched only among upregulated genes at 3dpS, and not 21dpS, included “ribosome” and “ribosome large subunit biogenesis” (Fig. S1E). These included the ribosome biogenesis factor *nsa2* and *gtpbp4* which is involved in biogenesis of the 60S ribosomal subunit, which were significantly upregulated at 3dpS but not 21dpS. However, the function “ribosomal large subunit assembly” was enriched among genes upregulated at 21dpS and not 3dpS. This included some genes that were only significantly increased at 21dpS such as *ruvbl1* and *srfbp1*, and others that were significantly increased at both 3dpS and 21dpS such as *rsl24d1*, *ptges3l*, and *gltscr2* (Table S3A). Overall, genes involved in ribosome biogenesis were induced more strongly at 3dpS compared to 21dpS, suggesting it is a relatively early response to starvation with aspects that continue through 21dpS (Fig. S3CD, Table S3A).

Also among the genes upregulated specifically at 3dpS was the heat shock protein *hsp90ab1*, a molecular chaperone previously shown to be upregulated in adult zebrafish liver in response to starvation [17]. The most significantly upregulated gene in our dataset was the enteropeptidase/enterokinase *tmprss15,* that converts trypsinogen into active trypsin which in turn activates pancreatic enzymes and potentially also antimicrobial proteins in the intestinal lumen (Fig. 3D-F) [63, 64]. Intestinal expression of *tmprss15* was not affected by starvation at 3dpS, but was upregulated 6-fold by 21dpS (Fig. 3D-F). Notably, a deficiency in *TMPRSS15* has been shown to confer a lean, starvation-like phenotype in humans, consistent with its known key role in nutrient digestion and absorption [65]. Upregulation of *tmprss15* in the starved zebrafish intestine suggests potential adaptive increases in nutrient digestion programs to salvage nutrients from the intestinal lumen, or in antimicrobial defense against an altered and potentially pro-inflammatory microbiome.

Similarly, functions enriched specifically among downregulated genes at 3dpS and not at 21dpS included “metabolism of lipids”, “regulation of cell proliferation”, and “ubiquitin-dependent protein catabolic process” (Fig. S1E). This included downregulation of the acyltransferase *lclat1* and fatty acid binding protein *fabp1b.1* at 3dpS but not 21dpS. However, related functions “glycerophospholipid metabolism”, “lipid transport”, and “lipid metabolic process” were enriched among downregulated genes at 21dpS but not 3dpS. These included the phospholipase *pla2g12b*, the fatty acid desaturase *fads2*, the lipid transfer protein *scp2a*, and multiple apolipoproteins including *apoa1a*, *apoa4b.1*, and *apobb.1* (Figs. 3D, S1E, S3AB) [66]. Notably, apolipoprotein genes have been shown to be downregulated in starved rainbow trout livers [67]. Although these genes were significantly downregulated only at 21dpS, most began trending towards downregulation at 3dpS. Yet other genes involved in these functions including *elovl2* and *fabp1b.1* were significantly downregulated at both 3dpS and 21dpS with a larger difference at 21dpS (Figs. 3D and S1A, Table S3A). Notably, the transporter *slc31a1*/*ctr1* involved in dietary copper uptake was also significantly downregulated at both timepoints. Thus, while shorter durations of starvation such as 3 days lead to a downregulation of some metabolic functions, most of the genes involved in lipid metabolism are not significantly downregulated until 21 days of starvation. Prolonged starvation therefore leads to reduced expression of genes involved in lipid biosynthesis and transport, perhaps representing an adaptation to the prolonged absence of dietary fats and other nutrients. However, this contrasts with shorter periods of starvation, such as 48 h, where other zebrafish studies have observed an increase in lipid catabolism, potentially to increase available energy and improve resistance to cold [68].

The genes significantly downregulated by starvation were also enriched for host immune functions. For example, the signal transducer *stat1b*, which is required for inflammatory responses in the intestine and for myeloid development in zebrafish [69, 70], and the interferon responsive gene *ifit8* are downregulated by 3dpS and continuing through 21dpS. By 21dpS, *ifit15* and the antiviral protein *rsad2* are also significantly downregulated. In accord, downregulated genes at 21dpS were enriched for functions involved in “defense response to virus”. Finally, the carboxypeptidase *cpa5* which is considered a marker for mast cells in zebrafish [71] was also significantly downregulated at 21dpS, suggesting a potential reduction in mast cell number or activity in the intestinal tissue. Although several immune-related genes were downregulated in starved fish, complement proteins *c6* and *c7* were both significantly upregulated in starved fish (Table S3). Our analysis of genes downregulated during starvation therefore suggests a reduction or impairment in immune function and inflammatory tone in the intestine during starvation, along with significant reductions in lipid metabolism and lipoprotein production. Reduced immune function during starvation may represent a mechanism contributing to the microbial community alterations observed at those timepoints.

While there were too few significant genes after refeeding at 3dpR to permit analysis of functional enrichment, several of these genes were suggestive of potential intestinal functions. This included increased expression at 3dpR of genes encoding the tandem-duplicated trypsin-like serine proteases *prss59.1* and *prss59.2*. This small set of genes also included three mitochondrial enzymes beta carotene dioxygenase-like gene *bco2l,* involved in cleavage of dietary carotenoids into retinoids towards Vitamin A synthesis; and dimethylglycine dehydrogenase *dmgdh*, involved in glycine synthesis and production of sarcosine in the choline oxidation pathway. Notably, *Dmgdh* was previously shown to be induced in mouse livers upon fasting, and reduced in the livers of ground squirrels preparing for hibernation [72, 73]. Of the 11 genes differentially expressed in 3dpR refed fish compared to fed controls, 6 were also differentially expressed at 21dpS including *prss59.1*, *prss59.2*, and *bco2l*. These may represent starvation adaptations that remain altered after restoration of feeding or that respond to both starvation as well as to refeeding.

Although we had already removed from this analysis any genes that were differentially expressed between fed control timepoints (Table S3B, Fig. S2), we wanted to further evaluate whether there were broader biological processes that may have been differential between those fed control samples that could affect our comparisons with starved/refed animals. We therefore performed GO term analysis of genes identified as significantly different between our fed control timepoints. The GO term “lipid metabolic process” was significantly enriched among genes that were significantly downregulated in 21dpS fed relative to 3dpS fed fish. Conversely, the GO terms “lipid localization” and “response to lipid” were significantly enriched among genes that were significantly upregulated in 21dpS fed relative to 3dpS fed fish (Table S3D). Importantly, the GO term “lipid metabolic process” was also enriched in genes that were significantly downregulated in 21dpS starved relative to 21dpS fed fish, even after genes that were significant in our control analysis were removed (Fig. S1E). This raised the possibility that our observed impacts of starvation on lipid metabolism genes here may be driven in part by unusually low expression of certain lipid metabolic genes in 21dpS fed fish, whereas other related lipid metabolic functions may be unusually high in 21dpS fed fish relative to the other fed timepoints. We therefore evaluated the log2 fold changes of genes from this control analysis alongside genes that were significantly different between starved and fed fish to discern if some of these differences may be driven by the control 21dpS fed fish (Fig. S3). We found that genes under the GO terms “ribosome” and “ribosome large subunit biogenesis” do not have differential expression in starved fish that is affected by unusual gene expression in the fed fish (Fig. S3C-D). In contrast, a subset of genes such as *pdk3b, syt1b, apoa1a, apoa4b.1,* and *fads2* which are significantly downregulated in starved fish at 21dpS relative to 21dpS fed, may be due in part to unusually high expression of these genes in 21dpS fed fish (Fig. S3A-B). However, most genes emphasized here, such as *elovl2, pla2g12b, slc31a1,* and many others, are not affected by abnormalities within the fed fish cohort and are likely true biological effects of the starvation treatment. To confirm this, we generated an independent cohort of adult zebrafish subjected to 21 days of starvation or fed normally and used quantitative RT-PCR to evaluate intestinal expression of lipid metabolism genes. In accord with our RNA-seq data from the original experimental cohort (Table S3), starved fish at 21dpS displayed significantly reduced expression of *elovl2, pla2g12b,* and *apoa4b.2* compared to 21dpS fed controls (Fig. S4).

### Several genes responsive to starvation are also responsive to high fat feeding

**Fig. 4:**
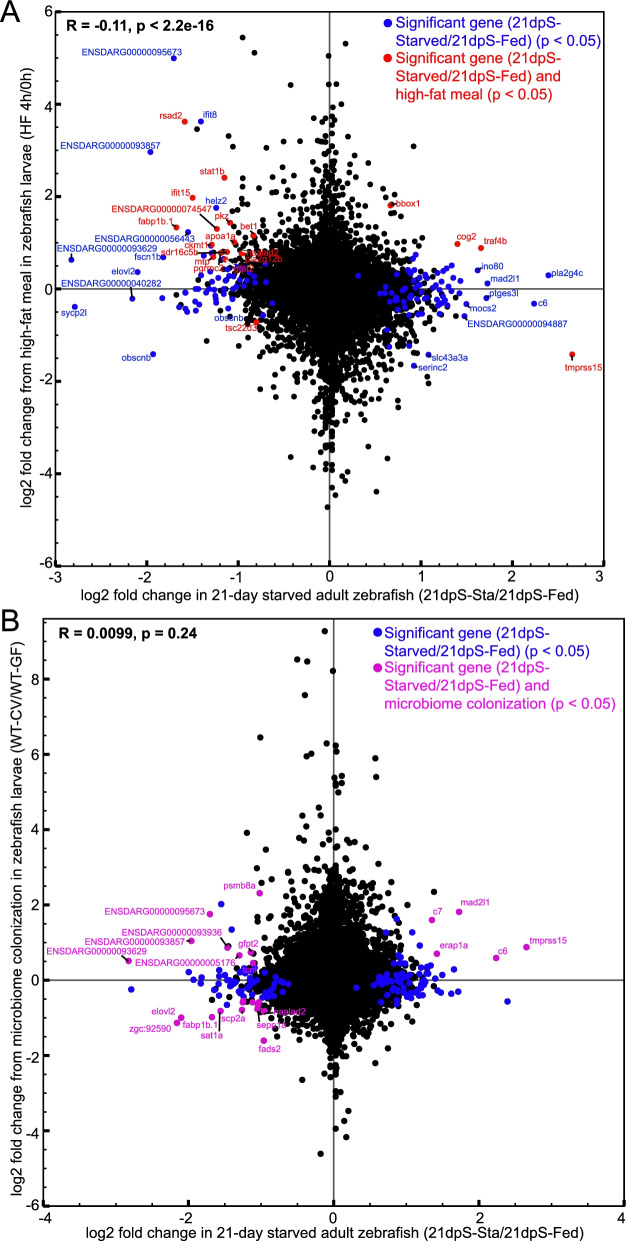
Some genes responsive to starvation in the intestine are also responsive to high fat feeding and microbial colonization. **A** Log2 fold changes for genes from 21dpS (X-axis) plotted according to their log2 fold changes in egg yolk-fed larval zebrafish compared to unfed controls (Y-axis), described in Zeituni et al [59]. Significantly differential genes only in starved zebrafish are plotted in blue, whereas genes significant in both datasets are plotted in red. Pearson’s correlation revealed a significant correlation between the two datasets (*p* < 0.05). **B** Log2 fold changes for genes from 21dpS (X-axis) plotted according to their log2 fold changes in zebrafish larvae colonized with a microbiome compared to germ-free controls (Y-axis), described in Davison et al [74]. Genes with significant log2 fold changes only in starved zebrafish are plotted in blue, whereas genes significant in both datasets are plotted in magenta. Pearson’s correlation did not reveal a significant correlation between the two datasets (*p* > 0.05)

To interpret which starvation-responsive genes from our dataset responded transcriptionally across a broad range of nutrient availability, and which ones may constitute a starvation-specific response, we referenced our intestinal RNA-seq results against previously published RNA-seq data comparing digestive tracts from zebrafish larvae that were either unfed or fed a high-fat meal (chicken egg yolk) [59]. This revealed a large overlap in significantly differentially-expressed genes (Fig. 4A). Particularly, genes involved in lipid transport and metabolism such as *fabp1b.1* and *pla2g12b* that were downregulated during starvation were upregulated during high fat feeding in zebrafish, underscoring the ability of these genes to respond to nutrients in zebrafish. Several genes involved in immune function such as *rsad2, stat1b,* and *ifit15* were downregulated during starvation, and were upregulated after high fat feeding. Also among the overlapping genes was the enteropeptidase *tmprss15* which was upregulated during starvation but downregulated by high fat feeding, and gamma butyrobetaine hydroxylase *bbox1* which which was upregulated by both starvation and high fat feeding.

While there was an overlap between genes in the above datasets that implicated them in the intestinal nutrient response, several genes that were significantly affected by starvation were not significantly affected by high fat feeding. These genes included the complement factor *c6*, the fatty acid elongase *elovl2,* and the phospholipase *pla2g4c*. These findings suggest that some classes of genes involved in lipid transport or inflammation may be differentially regulated by factors uniquely associated with starvation and not nutrient excess inherent to high fat feeding. Alternatively, these differences could be ascribed to transcriptional responses unique to zebrafish life stages (adult vs larvae) or organs (intestine vs complete digestive tract including intestine, liver, pancreas, and swim bladder), or to indirect effects of high fat egg yolk feeding that are unrelated to nutrition.

### A small subset of genes responsive to starvation are also responsive to microbial colonization

We and others have shown that intestinal gene expression is regulated in part by the presence and composition of the intestinal microbiome [55, 74–76]. Our 16S rRNA sequence data revealed that starvation induced marked and reversible alterations to gut microbiome composition including enrichment of *Vibrio* sp., members of which have been shown to be pro-inflammatory, and a decrease in similarity in microbiome composition between starved fish and their environmental samples [48]. Although these results suggest altered gut microbial ecology during starvation, our study design did not permit us to causally link our observed changes in intestinal transcriptome and microbiome. Therefore, in order to identify transcriptional responses to starvation that may also be sensitive to microbiome, we compared our RNA-seq data to a previous study investigating the effect of microbial colonization on larval zebrafish digestive tracts [74]. We found no correlation between the two datasets, implying that there may not be extensive overlaps between transcriptional responses to microbial colonization and starvation at these timepoints (Fig. 4B). This modest overlap may be due to transcriptional responses unique to zebrafish life stages (adult vs larvae) or organs (intestine vs complete digestive tract including intestine, liver, pancreas, and swim bladder). However, we did identify several overlapping genes that were significant in both datasets. Two complement factors, *c6* and *c7,* which were upregulated in colonized zebrafish, were also upregulated during starvation. In addition, two genes involved in the processing of major histocompatibility complex, *mad2l* and *erap1,* were also among the overlapping genes. A small set of genes involved in lipid metabolism and intracellular cholesterol transport, such as *elovl2*, *fads2*, *fabp1b.1* and *scp2a* were significantly downregulated by both colonization and starvation. The enteropeptidase *tmprss15*, which was differentially expressed during both starvation and high fat feeding, was also significantly upregulated by microbiome colonization. Overall, this comparison identified candidate genes that respond to both starvation and microbiome colonization.

### Starvation-responsive genes may also be controlled by the transcription factor hepatocyte nuclear factor 4 alpha (HNF4A)

**Fig. 5:**
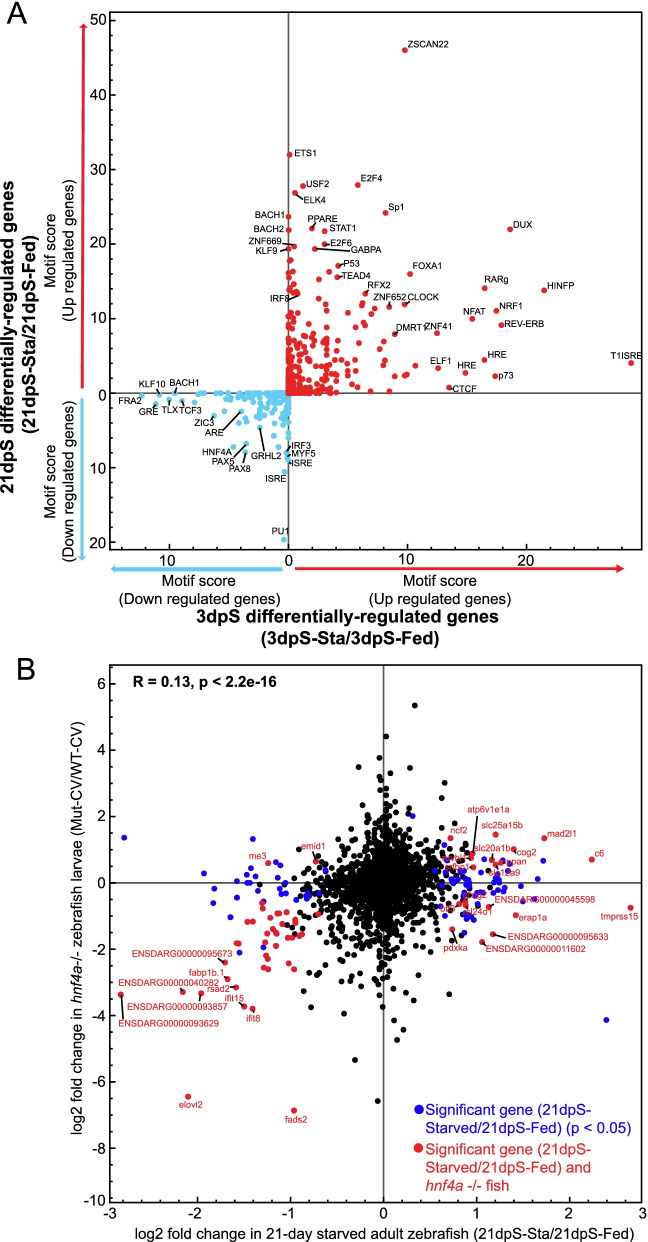
The transcription factor *hnf4a* may regulate a subset of genes involved in starvation. **A** Scatterplot for motif enrichment scores for genes that were significantly up- or down-regulated at 3dpS (X-axis) and 21dpS (Y-axis), according to HOMER analysis of transcription factor binding sites within 10 KB upstream or downstream of the genes’ transcription start sites at each time point, based on whether these sites were located within accessible chromatic regions. The motif score reflects the log10 *p*-value assigned by HOMER to each motif, comparing genes up- or down-regulated at the specified timepoint using as background the genes that were regulated in the opposite direction at the same timepoint. *HNF4A* is among the transcription factors whose binding sites are enriched at genes downregulated at both 3dpS and 21dpS. **B** Log2 fold changes for genes from 21dpS (X-axis) plotted according to their log2 fold changes in digestive tracts dissected from *hnf4a* mutant zebrafish larvae compared to wild-type controls (Mut-CV/WT-CV) (Y-axis), described in Davison et al [74]. Genes with genes with significant differential gene expression (21dpsSta/Fed) changes only in starved zebrafish are plotted in blue, whereas genes significant in both datasets are plotted in red. Pearson’s correlation revealed a significant correlation between the two datasets (*p* < 0.05)

We next sought to identify transcription factors putatively linked to the regulation of the starvation response. Using HOMER [77], we queried the genomic regions near all genes identified as significantly upregulated or downregulated by starvation. We restricted our search to regions within the gene body plus the flanking 10 kb upstream and downstream that we previously identified as accessible chromatin in the zebrafish intestine [51]. This revealed vertebrate transcription factor motifs significantly enriched near starvation responsive genes at either 3dpS or 21dpS (Fig. 5A; Table S3C). PAX5 motifs were significantly enriched near downregulated genes, while FOXA1 motifs were significantly enriched near upregulated genes, both at 3dpS and 21dpS. Both PAX5 and FOXA1 have been implicated in intestinal development in mice [78, 79]. Further, HNF4A motifs were significantly enriched near downregulated genes at both 3dpS and 21dpS. We previously showed that the nuclear receptor HNF4A mediates host transcriptional responses to microbial colonization in zebrafish [74]. In addition, HNF4A in other animals is required for intestine-specific gene regulation and has conserved roles in glucose homeostasis, gluconeogenesis, and lipid metabolism, indicating that starvation-linked genes may be under the control of HNF4A [80–83]. This observation suggested that HNF4 transcription factors might facilitate both responses to starvation and changes to host microbiome.

Based on these previous findings, a comparison of our starvation dataset to an RNA-Seq dataset from *hnf4a* mutant zebrafish [74] demonstrated that genes putatively controlled by *hnf4a* were significantly downregulated in our dataset (Fig. 5B). GO Term analysis revealed that “lipid metabolic process”, “viral response”, and a variety of other metabolic functions were significantly enriched among these overlapping genes. Specifically, the genes *pla2g12b* and *elovl2,* and other genes involved in lipid metabolism that were downregulated significantly in starved fish, were all downregulated in *hnf4a*^*−/−*^ fish, implying that lipid metabolic responses to starvation might be positively regulated by Hnf4a. Similarly, several of the immune response genes differentially regulated in starved fish such as *ifit15, ifit8, c6,* and *erap1a* were also differentially regulated in *hnf4a −/−* fish, suggesting that the immune response to starvation may also be partly influenced by Hnf4a function.

## Discussion

Interaction between the microbiome and host metabolism is known to occur in diverse pathophysiological contexts including starvation and malnutrition. However, few previous studies have simultaneously explored changes in host gene expression and microbiome composition as a function of starvation [84]. We focused here on the intestine as the animal’s primary interface with the gut microbiome and dietary nutrients. Our RNA-Seq data suggests that cytoplasmic translation, ribosomal genes, and ribosomal synthesis genes are upregulated in the zebrafish intestine early in starvation, whereas DNA repair, and vitamin and cofactor metabolism genes become upregulated at 21dpS. Similarly, some pathways significantly downregulated at 21dpS were distinct from those downregulated at 3dpS, with 21dpS including genes involved in antiviral response, arginine and proline metabolism, and glycerophospholipid metabolism, among others. The distinct functions encoded at 3dpS and 21dpS suggest different stages of starvation, as previously described in zebrafish liver [52]. In sharp contrast to previous studies in other organs, only a handful of genes were differentially expressed after refeeding in the zebrafish intestine, suggesting that the adaptive physiology displayed by the intestine during prolonged starvation is rapidly reversible after refeeding. In starved and refed zebrafish livers, upregulated genes are enriched for functions such as the TCA cycle, proteasome assembly, oxidative phosphorylation, and DNA replication and repair [52]. Similar compensatory mechanisms have been observed to accompany refeeding in cattle livers, as well as in salmon and trout muscle [85–87]. These results suggest that the intestine may be particularly plastic in its adaptation to starvation and refeeding compared to other organs such as muscle and liver.

To explore gene regulatory mechanisms underlying the intestinal response to starvation, we provide evidence that *hnf4a* may regulate a substantial number of these starvation-associated changes, expanding the already large number of physiologic functions associated with this gene. Considering that *hnf4a* activity is suppressed by the microbiome in zebrafish and mice [74], *hnf4a* may link alterations in the host microbiome and transcriptome during starvation. Future studies could test the impact of starvation on Hnf4a occupancy using ChIP-Seq, or on chromatin accessibility or histone modifications in the intestinal epithelium to identify cis-regulatory regions involved in coordinating the starvation response. Our data also provide numerous candidate genes that can be used in future experiments to explore the specificity, regionality, and regulation of the starvation responses in the zebrafish intestine.

Although our RNA-Seq data suggests many commonalities between the starvation response in zebrafish and other vertebrates, it also highlights unique ways in which the zebrafish intestine may adapt to long-term starvation. For example, we observed an induction of complement proteins, ribosomal proteins, and a downregulation of the antiviral response during starvation. This is in contrast to rainbow trout liver where starvation was reported to reduce expression of ribosomal proteins [67]. Meanwhile, genes significantly downregulated at 21dpS included several involved in the antiviral response. These pathways have not been previously reported in other animals in the context of starvation and thus could represent adaptive mechanisms unique to the zebrafish.

Starved zebrafish exhibited significantly reduced growth that was not fully recovered during a 21-day refeeding timeline. This suggests that full somatic recovery from starvation may require more time, or that there are permanent somatic changes associated with starvation. In contrast, we find that the changes that starvation induces in the zebrafish intestinal transcriptome and microbiome are rapidly normalized after refeeding. Whereas starvation significantly affected the expression of over 200 unique genes in the intestine compared to fed controls, refeeding for just 3 days restored normal levels of expression for all but 9 genes (Fig. 3B-C, Fig. S2D). Similarly, intestinal microbial communities subjected to starvation displayed significantly increased diversity (Fig. 1D) and altered composition (Fig. 2A) compared to fed controls, yet those differences were largely normalized within 1 to 3 days of refeeding. By comparison, another animal that undergoes prolonged starvation, the hibernating ground squirrel, maintained baseline levels of intestinal microbiome diversity during early stages of winter hibernation, reduced diversity later in the winter, and then increased diversity upon refeeding in the spring [88]. That boost in diversity upon refeeding was attributed to new bacterial taxa associated with the introduced food. For this study, no samples of food-associated bacterial taxa were taken, so we cannot distinguish between these two possible explanations. Regardless, the distinct effects of starvation and refeeding on intestinal microbiome diversity in zebrafish and ground squirrels underscores the importance of studying the ecology and physiology of prolonged starvation and refeeding in diverse animal hosts.

Our results also provide insight into the specific bacterial lineages that are most sensitive to starvation and refeeding in the zebrafish intestine. We previously demonstrated that *Vibrio* and *Plesiomonas* genera are part of a core gut microbiome of zebrafish [89]. We speculate that the opposing changes in relative abundance of these two taxa likely reflect differing abilities to survive in the altered environment of the starved gut (Fig. 2B, C, E). *Vibrio* sp. are common members of the intestinal microbiome in zebrafish, and their relative abundance correlates positively with intestinal inflammation [41, 42, 48]. This suggests that starvation-induced alterations in the relative abundance of *Vibrio* sp. and other bacteria in the zebrafish intestine might be linked with alterations in intestinal gene expression. During starvation in chickens, intestinal mucus is known to increase in abundance and thickness, possibly creating a competitive advantage for mucin-degrading bacteria [90, 91]. *Vibrio* spp. can degrade intestinal mucus, which may be why there is an observed increase in *Vibrio* during starvation [92, 93]. Conversely, *Plesiomonas* may be less suited for survival during prolonged starvation periods within the gut. It remains unclear if these changes in relative abundance were accompanied by alterations in microbial community density, which could be explored in future studies. It is striking that these and other starvation-induced perturbations to gut microbiome composition, similar to host gene expression in the gut, were largely restored within 1 to 3 days after refeeding. This underscores remarkable plasticity in intestinal physiology and microbial ecology in response to starvation and refeeding.

## Methods

### Animal husbandry

Unless otherwise stated, all fish were maintained on a 14-h light cycle at 28 °C. All zebrafish used for these experiments were born on the same day from 1 (RNA-Seq) or 3 (16S rRNA gene amplicon sequencing and qRT-PCR assays) breeding pairs from a single sibship.

Animals used for RNA-Seq and 16S rRNA gene amplicon sequencing were generated at Duke University as follows. Fertilized embryos were transferred into Petri dishes containing egg water (6 g sea salt, 1.5 g calcium sulfate, 0.75 g sodium bicarbonate, 10–12 drops methylene blue, 10 L water) at a density of 50 embryos/dish incubated at 28.5 °C. At 1-day post-fertilization (dpf), embryos were transferred to 3 L tanks containing 500 mL water from a recirculating zebrafish aquaculture system (system water). Each tank contained 10 (16S rRNA gene sequencing) or 30 (RNA-Seq) embryos. Fish were then maintained under standard husbandry on a recirculating zebrafish aquaculture system until the start of the experiment at 60dpf. Zebrafish were then randomly transferred into four (RNA-Seq) or eight (16S rRNA gene sequencing) clean 10 L tanks at a density of 44 (RNA-Seq) or 67 (16S rRNA gene sequencing) fish per tank, with half the tanks receiving no food for the following 21 days (Fig. 1A). Following the 21 days of starvation, feedings for all tanks were allowed to occur as per standard husbandry: two feedings of live Artemia per day interspersed with two feedings of Gemma 300 (Skretting). Over the 21 days of starvation and 21 days of refeeding, all fish remained on the recirculating zebrafish aquaculture system and we observed no mortality in any condition or experiment.

Animals used for qRT-PCR validation were generated at the Carnegie Institution as follows. Fertilized embyros were transferred at 5dpf to 10 L tanks, with each tank containing approximately 70 larvae. Fish were fed a regimen of Gemma Micro (Skretting) with gradually increasing pellet sizes. For the first 2 weeks of feeding, larvae were fed exclusively Gemma Micro 75, and then transitioned to a diet of Gemma Micro 150 supplemented with live Artemia (www.artemia-international.com) for the subsequent 3 weeks, at which point fish were transitioned to anadult diet of Gemma Micro 300 with only occasional live brine supplementation. Adults raised in this manner were transferred to one of 12 experimental 3 L tanks that were balanced for density and gender ratio, and half of those tanks received no food for the following 21 days.

All fish to be sampled on a particular day were collected prior to the first daily feeding in the fish facility. Samples for 16S rRNA gene amplicon sequencing were collected at 0 days post-starvation (0dpS), 1 dpS, 3dpS, 7dpS, 21dpS, 1 day post-re-feed (dpR), 3dpR, 7dpR, and 21dpR (Fig. 1A) with six randomly selected fish at each time point per tank were euthanized by tricaine overdose (0.83 mg/ml tricaine). Fish were imaged on a dissecting scope to facilitate subsequent standard length (SL) and height at anterior of anal fin (HAA) measurements [94]. Intestinal tracts were then dissected from each fish and placed individually in lysis buffer (20 mM Tris-HCl (pH 8.0), 2 mM EDTA (pH 8.0), 1% Triton X-100, flash-frozen in a dry-ice/ethanol bath, and stored at − 80 °C until DNA extraction.

Samples for RNA-Seq were collected at 3dpS, 21dpS, and 3dpR. At each time point, three randomly selected fish per tank were euthanized by tricaine overdose (0.83 mg/ml tricaine). Fish were imaged on a dissecting scope to facilitate subsequent standard length (SL) [94]. Intestinal tracts were then dissected from each fish and placed individually in 2 mL cryovials filled with TRIzol reagent (Thermo Fisher, 15,596,026), flash-frozen in a dry ice-ethanol bath, and stored at − 80 °C until RNA extraction.

Samples for qRT-PCR were collected at 21dpS. Fish were euthanized by tricaine overdose, and their intestines were dissected and stored individually in 0.5 mL of RNAlater.

### 16S rRNA gene sequencing

Genomic DNA was extracted from individual zebrafish intestinal tracts using Qiagen DNeasy Blood and Tissue Kits (Qiagen, modified with bead-beating as previously described) [42]. Genomic DNA was subsequently used as template for PCR amplification of the v4 region of 16S rRNA gene and 150 bp/cycle paired-end sequencing was performed on an Illumina HiSeq 2000 Sequencing System (see Table S4 for primers) at the University of Oregon Genomics and Cell Characterization Core Facility.

### 16S rRNA gene sequence bioinformatic and statistical analysis

FASTQ files were demultiplexed and split by sample ID using QIIME (v1.9.1). Within RStudio version 3.4.1, the files were then quality filtered, trimmed, denoised, merged, checked for chimeras, and assigned taxonomy using DADA2. Taxonomic assignments were made using the Silva v132 database. Data analysis used the R packages vegan and phyloseq. LEFsE was accessed through the Huttenhower Galaxy website: https://huttenhower.sph.harvard.edu/galaxy/. The following packages were used for analyses and the creation of figures: ape, picante, vegan, ggtree, SEPP, plotyly, heatmaply, and pairwiseadonis [95–102].

### RNA extraction and sequencing

Frozen whole intestinal samples stored at − 80 °C were homogenized using Zirconium oxide beads (Biospec, 11,079,107) and a Vortex Genie2 (Scientific Industries, 1311-V) fitted with a Vortex Adapter (Scientific Industries, 13,000-V1–24) in three 45-s intervals. Samples were put on ice in-between homogenization to prevent overheating. Following homogenization, a phase separation was performed by adding 200ul of chloroform to each sample and mixing by vigorous inversion 15 times. Samples were then incubated at room temperature for 3 min and centrifuged at 12000rcf for 15 min at 4 °C. 500ul of the aqueous upper phase from each sample was then transferred to a new Eppendorf tube, to which 500uL 70% Ethanol in DEPC water was added and vortexed. Following phase separation, samples were DNase treated and total RNA was extracted via column purification using the PureLink DNase Set (Thermo Fisher, 12,185,010) and the PureLink RNA Mini kit (Thermo Fisher, 12,183,025) according to the manufacturer’s instructions. Final sample quality and concentration were assessed via spectrophotometry and samples were stored at -80 °C until submission to the Duke Sequencing and Genomic Technologies Core. RNA-seq libraries were prepared and sequenced by Duke Sequencing and Genomic Technologies Core on an Illumina HiSeq 2500 to generate 50 bp single-end reads (SR).

### RNA-seq bioinformatics

All raw zebrafish RNA-seq data was processed on the Galaxy server [103]. Raw fastq files were trimmed using Trim Galore [104]. Trimmed fastq files were then mapped to the zebrafish genome (GRCz10) using STAR using default settings to generate BAM files, which were converted to counts using HTSeq (v.0.9.1). BAM files were converted to bigWig files using the bamCompare tool (v2.5.0) with RPKM normalization before visualization on the UCSC Genome Browser [105–107].

TPM expression values were obtained for transcripts via Salmon [108]. Pairwise differential gene expression tests were carried out with DESeq2 using counts files generated by HTSeq [108, 109]. For comparisons between starved and fed fish, the default significance threshold of adjusted *p*-value 0.05 was used for each comparison. For comparisons across fed fish controls, the significance threshold was defined as the gene either having an absolute log_2_ fold change greater than 1.0 or a *p*-value less than 0.05 (See Figs. S1, S3, and Table S3B). Versions of the analysis that include the genes removed by this filtering step are available in Fig. S2.

Hierarchical clustering of log_2_ fold change values for genes was performed using Cluster 3.0, and heat maps were generated using Java Treeview [110, 111].

HOMER software (http://homer.ucsd.edu/homer/motif/) analysis was performed on significantly upregulated and downregulated genes at both 3dpS and 21dpS (3dpS starved/3dpS fed and 21dpS starved/21dpS fed, respectively), using regions within the gene body plus the flanking 10 kb upstream and downstream that we previously identified as accessible chromatin in the zebrafish intestine [51] using the findMotifs.pl command. A motif score was obtained by taking the -log_10_ values of the *p*-values assigned by HOMER. A motif was then deemed ‘enriched’ amongst either upregulated or downregulated genes at each timepoint (3dpS or 21dpS) based on whether it had a higher motif score among the upregulated or downregulated gene sets.

For comparisons with the larval zebrafish egg yolk feeding dataset, log_2_ fold changes in 21dpS fed fish relative to 21dpS starved were compared to log_2_ fold changes in larval zebrafish digestive tracts 4 h after egg yolk feeding (i.e. “HF 4h logFC”) obtained from Supplementary Table 1 in [59], the raw data for which is available at accession GSE87704. For comparisons with *hnf4a* mutant and microbially colonization datasets, data was obtained from Supplemental Table 2 in [74], using log_2_ fold changes comparing digestive tracts from *hnf4a* homozygous mutant and wild-type 6dpf zebrafish larvae raised under conventionalized ex-germ-free conditions (“MutCV/WTCV”) and from wild-type 6dpf zebrafish larvae reared under germ-free of ex-germ-free conventionalized conditions (“WTGF/WTCV”), respectively. Raw data from [74] is available at accession GSE90462.

### Quantitative RT-PCR assays

Dissected intestines from 16 starved and 15 fed 5–6-month-old fish were stored in RNAlater and shipped to the Rawls lab. Tissue was homogenized with bead beater tubes (Bertin Corp, P000912-LYSK0-A) and RNA was extracted with chloroform, precipitated with isopropanol, washed with ethanol, and treated with DNase (Invitrogen, AM1906). RNA concentration was assessed using Qubit RNA BR Assay Kit (ThermoFisher, Q10210). 800 ng of RNA were then reverse transcribed using the iScript cDNA Synthesis kit (Bio-Rad, 1708891). Quantitative real-time PCR was performed on QuantBio Studio6 (Applied Biosystems) using gene-specific primers for *pla2g12b* (F 5′-CAGTACCGCTGCAGATATGGT-3′, R 5′- ATTCGGTACCTGGAAGCCAAG-3′), *elovl2* (F 5′- ACAGTTTTCAGCTGTCCCGT-3′, R 5′- CATCCTCTCACGCGGGTATC-3′), *apoa4b.2* (F 5′-TTGTGGTCTTTGCACTTGCT-3′, R 5′- TCATCTTGACGGTTTCCTCTG-3′) and *ef1a* (F 5′- CTTCTCAGGCTGACTGTGC-3′, R 5′- CCGCTAGGATTACCCTCC-3′). 6 samples (3 male, 3 female) in each group were randomly chosen and analyzed in duplicate 25 μl reactions using 2X SYBR Green SuperMix (PerfeCTa, Hi Rox, Quanta Biosciences, 95055) and normalized to the expression of *ef1a* as a house-keeping gene. Expression profile and associated statistical parameters were determined using the ∆∆CT method and graphed in Prism (GraphPad).

## Supplementary Information

**Additional file 1.**

**Additional file 2.**

**Additional file 3.**

**Additional file 4.**

**Additional file 5.**

## Data Availability

All quality filtering parameters for generating the sequence variants, ASV table and figures for the 16S rRNA analysis can be found at: https://github.com/alexmccumber/fishguts. The raw 16S rRNA gene amplicon FASTQ files can be accessed from the European Nucleotide Archive under project access number PRJEB31503. Raw and processed RNA-Seq data is available on NCBI GEO at the accession GSE140821.
