# Supplementary material for: Starvation causes changes in the intestinal transcriptome and microbiome that are reversed upon refeeding
Source: BMC Genomics. 2022 Mar 22;23:225. doi: 10.1186/s12864-022-08447-2 (PMC8941736; doi:10.1186/s12864-022-08447-2)
Supplement: Supplementary file 5 — Additional file 5. [file 12864_2022_8447_MOESM5_ESM.docx]

**Starvation causes changes in the intestinal transcriptome and microbiome that are reversed upon refeeding**

Jayanth Jawahar*^1^, Alexander W. McCumber*^2^, Colin R. Lickwar^1^, Caroline R. Amoroso^3^, Sol Gomez de la Torre Canny^1^, Sandi Wong^1^, Margaret Morash^1^, James H. Thierer^4,5^, Steven A. Farber^4,5^, Brendan J. M. Bohannan^6^, Karen Guillemin^7^, and John F. Rawls^1**^

^1^ Department of Molecular Genetics and Microbiology, Duke Microbiome Center, Duke University School of Medicine, Durham, NC 27710, USA

^2^ Department of Civil and Environmental Engineering, Duke University, Durham, NC 27708, USA

^3^ Department of Evolutionary Anthropology, Duke University, Durham, NC 27708, USA

^4^ Department of Embryology, Carnegie Institution for Science, Baltimore, MD 21218, USA

^5^ Department of Biology, Johns Hopkins University, Baltimore, MD 21218, USA

^6^ Institute of Ecology and Evolution, University of Oregon, Eugene, OR 97403, USA

^7^ Institute of Molecular Biology, University of Oregon, Eugene, OR 97403, USA

* authors contributed equally

**Correspondence to [john.rawls@duke.edu](mailto:john.rawls@duke.edu)

**Supplemental Figures 1, 2, 3, and 4**


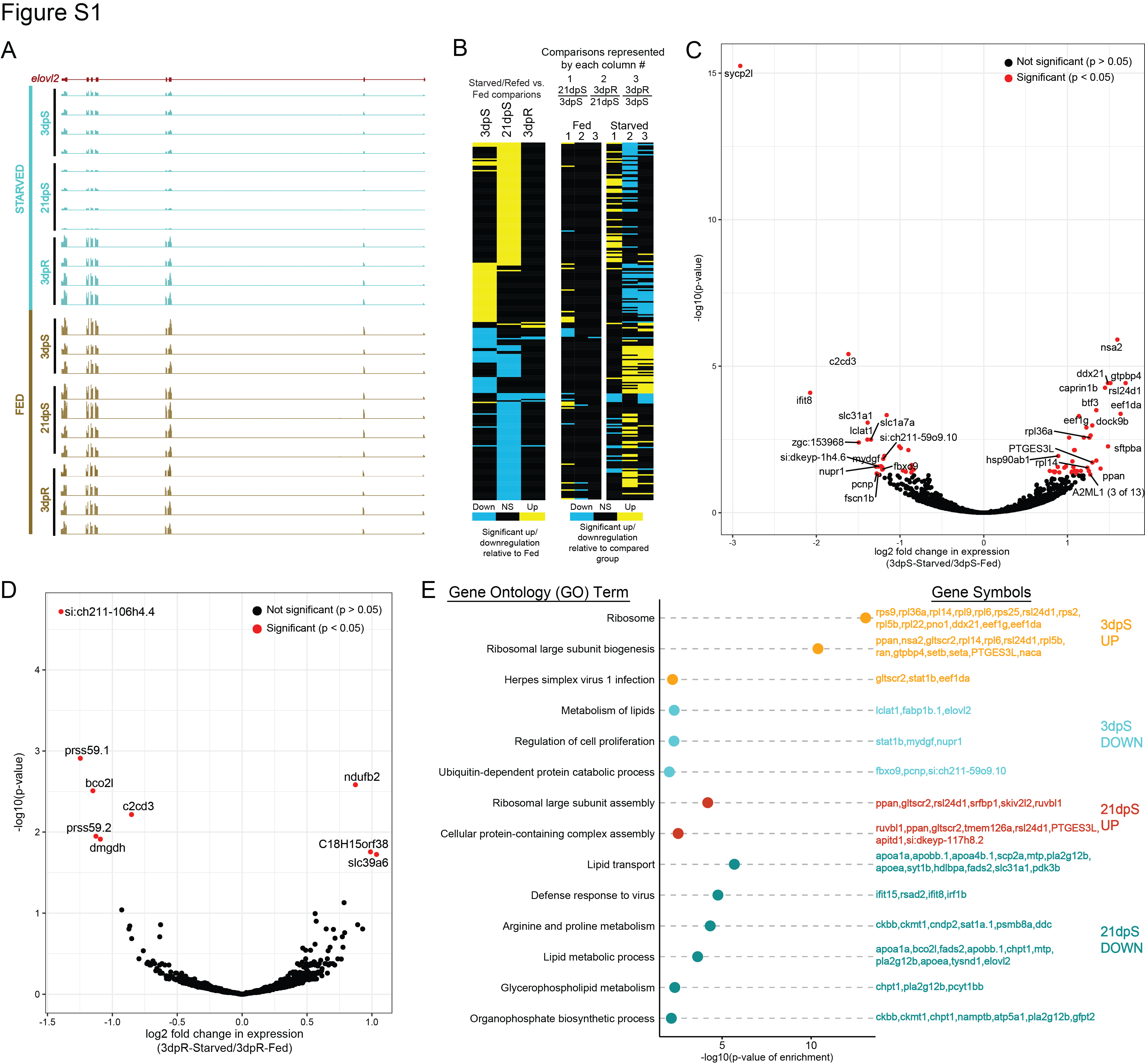


**Supplemental Figure 1: Starvation and refeeding in zebrafish cause alterations in intestinal gene expression.**

(A) UCSC tracks of all RNA-Seq samples show that *elovl2* mRNA, encoding a fatty acid elongase, is upregulated in starved fish and returns to levels comparable to the fed group upon re-feeding across all replicates.

(B) Hierarchical clustering of flattened values of log_2_ fold changes in gene expression in starved fish, seen in Figure 3C, alongside log_2_ fold changes of the same genes when compared across the same treatment group. These analyses suggested that the expression of very few genes were different in the fed control group across the study. Genes determined to be significantly differentially expressed in one or more of the fed control comparisons were removed from further analyses (removed genes listed in Table S3B).

(C) Log_2_ fold changes in gene expression in starved fish at 3dpS when compared to fed fish plotted according to their adjusted log_10_ p-values. (D) Log_2_ fold changes in gene expression in starved fish at 3dpR when compared to fed fish plotted according to their adjusted log_10_ p-values. Very few genes were significantly differentially expressed at 3dpR, suggested that refeeding returns many starvation-associated genes to their fed levels.
(E) Metascape analyses of Gene Ontology (GO) terms enriched among genes that are significantly differentially expressed at each timepoint, sorted according to upregulation and downregulation.


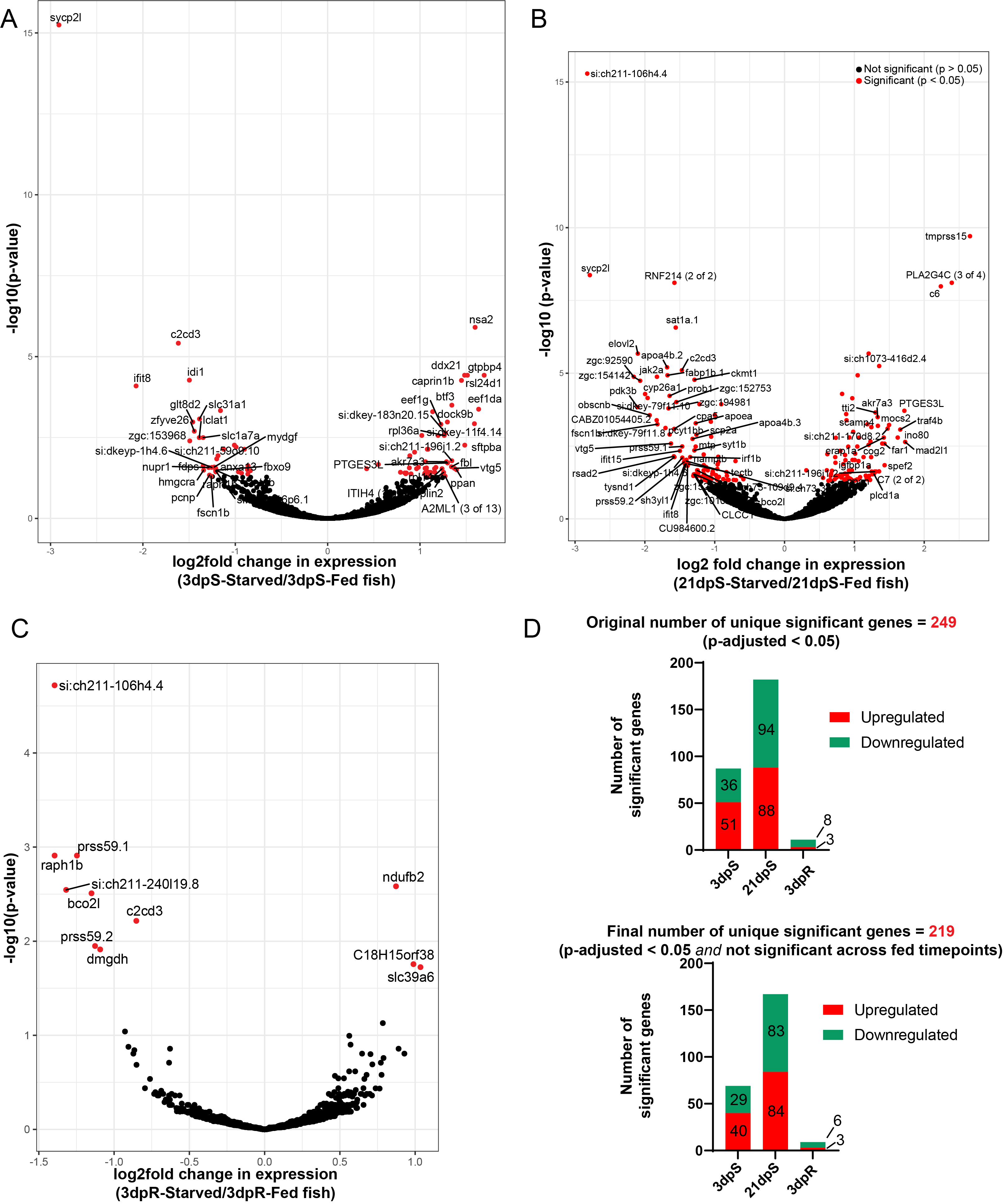


**Supplemental Figure 2: Significantly differential genes in starved vs. fed fish at three different timepoints, including genes that were significantly differential within fed fish.** As explained in Results, genes identified as significantly differentially expressed between any of the fed control timepoints were removed from analyses shown in Figures 3C, 4, 5, and S1. This supplemental Figure S2 is included to show results from the original gene list prior to their removal. Data shown in this supplemental Figure S2 includes all genes that were significantly differential in any of the starved vs. fed comparisons with an adjusted p-value less than 0.05, without removal of genes that showed significant changes in expression between fed timepoints.

(A) Log_2_ fold changes in gene expression in starved fish at 3dpS when compared to fed fish plotted according to their adjusted log_10_ p-values.

(C) Log_2_ fold changes in gene expression in starved fish at 21dpS when compared to fed fish plotted according to their adjusted log_10_ p-values.

(D) Log_2_ fold changes in gene expression in starved fish at 3dpR when compared to fed fish plotted according to their adjusted log_10_ p-values.
(E) Barplots showing the number of significantly differential genes between starved and fed fish at each timepoint with all genes having an adjusted p-value < 0.05 included (top), as well as the subset of genes that were not significantly differential within fed fish (bottom). The 219 gene set plotted on the bottom are those analyzed further in Figures 3C, 4, 5, and S1. Because some genes were significant in multiple timepoints, the total number of unique significant genes shown in red in each panel is smaller than the sum of significant genes across timepoints.


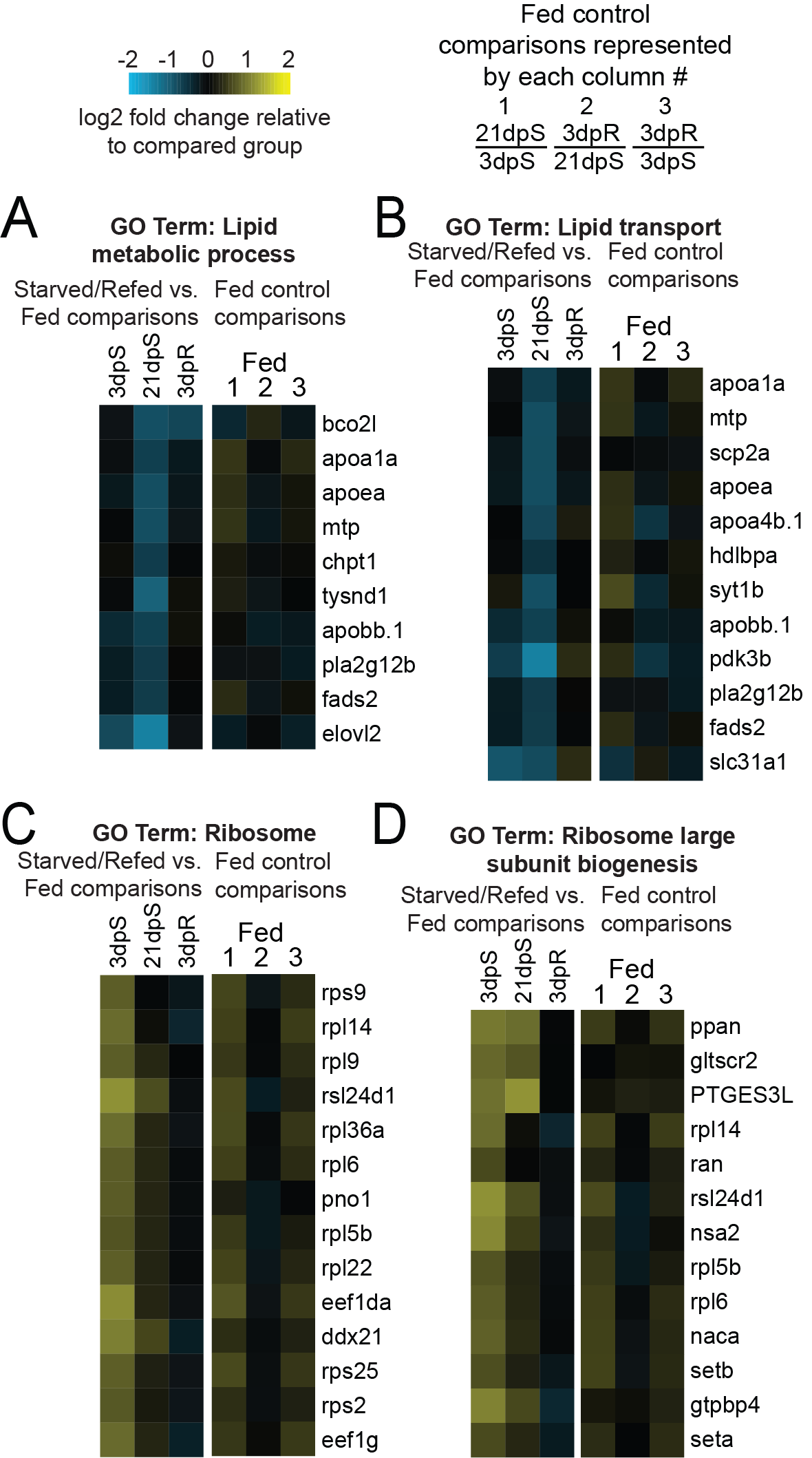


**Supplemental Figure 3: Differential gene expression observed in Starved/Refed vs. Fed comparisons and in Fed control comparisons.**

(A) Hierarchically clustered heatmap of (left) log_2_ fold changes (Starved/Refed vs. Fed control) fish shown for the genes under GO term “Lipid metabolic process”. On the right, a heatmap of the same genes is shown with their log_2_ fold changes within the fed control comparisons. None of the genes shown in panels A-D were significantly differential in fed control comparisons. Significance for the fed control comparisons is defined as either an absolute log_2_ fold change greater than 1.0 or a p-value less than 0.05.

(B) Hierarchically clustered heatmap of (left) log_2_ fold changes (Starved/Refed vs. Fed control) fish shown for the genes under GO term “Lipid transport”. Again, although none of the genes in the control comparisons shown here were significant, the log_2_ fold change in columns 1 and 2 suggests higher expression for some genes (e.g., *apoa1a, fads2, pdk2b, syt1b, apoa4b.1*) in 21dpS fed fish relative to the other two timepoints. For these few genes, lower log_2_ fold changes for these genes seen in 21dpS starved fish relative to 21dpS fed fish may be driven by higher expression in the 21dpS fed fish, and not necessarily related to their starvation treatment.

(C) Hierarchically clustered heatmap of (left) log_2_ fold changes (Starved/refed vs. Fed control) fish shown for the genes under GO term “Ribosome”.

(D) Hierarchically clustered heatmap of (left) log_2_ fold changes (Starved/refed vs. Fed control) fish shown for the genes under GO term “Ribosome large subunit biogenesis”.

**
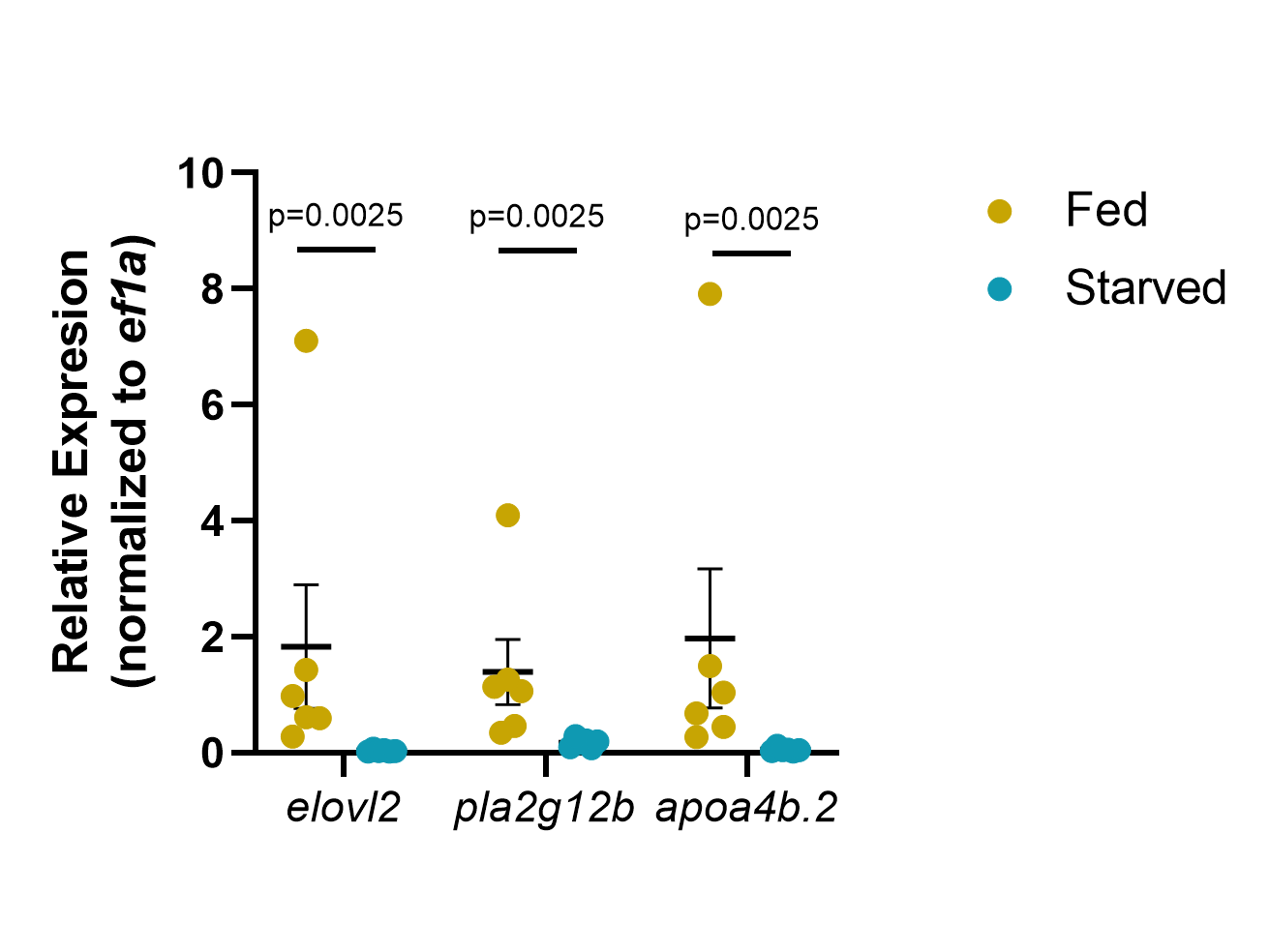
**

**Supplemental Figure 4: Quantitative RT-PCR validation assays confirm impact of starvation on lipid metabolism genes.** Intestines dissected from adult zebrafish starved for 21 days (Starved; n=6 animals) or fed normally (Fed; n=6 animals) were used to measure RNA levels of *elovl2*, *pla2g12b*, and *apoa4b.2* normalized to *ef1a*. Results are shown as mean with error bars representing standard error of the mean. P-values represent results of Mann-Whitney U test.
